# Tissue-Specific Expression Pattern in *Ancherythroculter nigrocauda*, a Sexually Size Dimorphic Fish

**DOI:** 10.3389/fgene.2021.777581

**Published:** 2021-12-08

**Authors:** Yanhong Sun, Huijie Wei, Jian Chen, Pei Li, Qing Yang, Guiying Wang, Qing Li

**Affiliations:** ^1^ Fisheries Research Institute, Wuhan Academy of Agricultural Sciences, Wuhan, China; ^2^ Wuhan Xianfeng Aquaculture Technology Co., Ltd., Wuhan, China; ^3^ Key Laboratory of Ecological Impacts of Hydraulic-Projects and Restoration of Aquatic Ecosystem of Ministry of Water Resources, Institute of Hydroecology, Ministry of Water Resources and Chinese Academy of Sciences, Wuhan, China

**Keywords:** sexual size dimorphism, comparative transcriptomics, tissue-specific expression patterns, RNA-seq, differentially expressed genes, WGCNA

## Abstract

Certain members of the Actinopterygii class are known to exhibit sexual dimorphism (SD) that results in major phenotypic differences between male and female fishes of a species. One of the most common differences between the two sexes is in body weight, a factor with a high economic value in aquaculture. In this study, we used RNA sequencing (RNA-seq) to study the liver and brain transcriptomes of *Ancherythroculter nigrocauda*, a fish exhibiting SD. Females attain about fourfold body weight of males at sexual maturity. Sample clustering showed that both sexes were grouped well with their sex phenotypes. In addition, 2,395 and 457 differentially expressed genes (DEGs) were identified in the liver and brain tissues, respectively. The gene ontology (GO) and Kyoto Encyclopedia of Genes and Genomes (KEGG) pathway enrichment analyses predicted the association of PPAR signaling, cytochrome P450, and steroid hormone biosynthesis to the differences in sexual size. In addition, weighted gene co-expression network analyses (WGCNA) were conducted, and the green module was identified to be significantly correlated with sexual size dimorphism (SSD). Altogether, these results improve our understanding of the molecular mechanism underlying SSD in *A. nigrocauda*.

## 1 Introduction

Sexual dimorphism (SD) is a common phenomenon exhibited by several members of the fish class Actinopterygii (Mei and Gui, 2015). One of the most common types of SD is sexual size dimorphism (SSD), i.e., one of the sexes has a relatively large body size—a characteristic that has a great economic value for aquaculture (Mei and Gui, 2015). SSD has been reported in more than 20 fishes, including olive flounder (Ryu et al., 2020), Japanese flounder (Yoneda et al., 2007), and Nile tilapia (Beardmore et al., 2001). The size of the female fish exhibiting SSD is evolutionarily linked to fecundity, whereas that of a male displaying SSD is linked to male–male competition (Parker, 1992). *Ancherythroculter nigrocauda* exhibits female-biased SSD, wherein the female *A. nigrocauda* grows considerably faster than males. Several studies have attributed this difference in growth between sexes to the influence of sex chromosomes in different species (Lindholm and Breden, 2002; Saether et al., 2007); however, these results lack a common pattern. In addition, increasing evidence supports the function of autosomes in SSD (Mank et al., 2007; Poissant et al., 2008). These discordances imply a complex mechanism governing SSD in fish than that proposed by current models.

*A. nigrocauda* is an aquaculture fish with high economic importance in China. Its breeding selection in the past decades has successfully fostered its body weight on sexual maturity from about 100 to 500 g (Unpublished data). A sexually mature female can grow to a body weight of about fourfold compared to a sexually mature male. Growth, a polygenic trait, depends on the interaction among environmental, nutritional, and genetic factors in fish (Fu et al., 2019). Fish growth is genetically controlled by the expression of growth hormone/insulin-like growth factor (*GH/IGF*) core genes that regulate the hypothalamus–pituitary–gonadal (HPG) axis and the subsequent activation of the Jak2/Stat5b pathway *via* a GH/GHR signal (Li and Lin, 2010; Rotwein, 2012). In zebrafish, Stat5b regulates sexually dimorphic gene expression in the liver (Huang et al., 2018). Although certain studies have been conducted in fishes, it is still difficult to uncover the genetic network regulating SSD (Wang et al., 2018a; Ryu et al., 2020).

The liver is a critical regulator of metabolic homeostasis because it integrates sex hormone signals triggered by alterations in metabolism and physiological state (Toews et al., 2021). Sex hormones control the body’s growth by influencing the release of GH and its activity (Ryu et al., 2020). As an important upstream regulator, the brain regulates the reproductive and somatotropic axes by secreting different neuroendocrine factors. In this study, we used the transcriptome data from liver and brain tissues to explore the genetic network regulating SD in *A. nigrocauda*. The core differentially expressed genes (DEGs) were identified and their effect on the growth rate was studied. The expression data from different tissues can provide us a better understanding of the function and influence of the liver and brain on SSD.

## 2 Methods

### 2.1 Samples Used

In this study, six female and six male fish aged 18 months were collected from the fishing farm in the Institute of Aquaculture, Wuhan Agriculture Academy (114.24 N, 30.34 E). These were reared in the same pond. Their body weights are shown in Table 1; their sex was confirmed by studying the mature gonads. Before sacrifice, all fish were anesthetized with MS222 (Sigma, United States). Six liver and six brain tissues from both sexes (a total of 24 samples) were collected and placed in liquid nitrogen to extract the total RNA.

**TABLE 1:** Summary of sequencing and mapping statistics for 24 samples used in the study.

Sample ID	Raw reads	Filtered reads	Filter ratio	Uniquely mapped reads	Uniquely mapped reads’ ratio	Overall alignment rate
s17B_L	25921109	24560619	94.75	19503215	79.41	86.74
s18B_L	27638696	26161055	94.65	20763897	79.37	87.14
s19B_L	24878281	23547120	94.65	17777227	75.5	83.31
s20B_L	24357231	23183480	95.18	18576145	80.13	87.65
s23B_L	27173846	25491877	93.81	20287459	79.58	86.93
s25B_L	21822969	20717450	94.93	16200317	78.2	85.65
s2B_H	27788628	26270907	94.54	20478581	77.95	85.52
s4B_H	24543447	22937404	93.46	16889132	73.63	82.44
s5B_H	24918061	23544275	94.49	17432806	74.04	82.38
s6B_H	25432716	23784897	93.52	17057381	71.72	80.09
s7B_H	23773852	22178050	93.29	17401114	78.46	86.05
s9B_H	22208433	20949070	94.33	15388120	73.45	80.84
s17L_L	30207759	28720121	95.08	20918986	72.84	89.67
s18L_L	27327905	25719985	94.12	18999102	73.87	91.69
s19L_L	26390067	25079004	95.03	18262377	72.82	89.63
s20L_L	25680881	24232304	94.36	17628822	72.75	90.24
s23L_L	30102767	28610142	95.04	21339427	74.59	90.06
s25L_L	28631285	26862570	93.82	19873907	73.98	90.3
s2L_H	29599332	27718217	93.64	19846083	71.6	87.88
s4L_H	26735365	24996197	93.49	17015259	68.07	84.59
s5L_H	25989617	24353890	93.71	17333415	71.17	89.86
s6L_H	20942980	19656506	93.86	13913579	70.78	87.57
s7L_H	24780632	23483490	94.77	16776516	71.44	88.04
s9L_H	21274486	20085238	94.41	14381774	71.6	88.07

All fish experiments were performed according to the guidelines established by Wuhan Agriculture Academy. All fish were treated humanely and ethically, and all experimental procedures were approved by the Wuhan Agriculture Academy.

### 2.2 RNA Extraction and Library Construction

The total RNA from the liver and brain tissues was extracted using the SV Total RNA Isolation System (Promega, United States) according to the manufacturer’s instructions. Next, the RNA concentration and purity were evaluated using NanoDrop 2000 (Thermo Fisher Scientific, United States). Finally, an Agilent Bioanalyzer 2,100 (Agilent Technologies, United States) was used to assess the integrity of RNA, and only samples with RNA integrity number (RIN) greater than 7 were used to construct the libraries.

The libraries were constructed using a TruSeq Stranded mRNA Prep Kit (Illumina, United States) according to the manufacturer’s instructions. The libraries were sequenced by Biomarker Technologies (Beijing, China) on the Illumina NovaSeq platform using the PE150 strategy. The raw sequencing data were deposited in the NCBI Sequence Read Archive (SRA) under accession numbers PRJNA762193.

### 2.3 Raw Read Filtering, Genome Mapping, and DEG Identification

Trimmomatic v0.3.8 (Bolger et al., 2014) was used to trim the raw reads and remove the adapters and low-quality bases. In addition, leading and trailing sequences with quality lower than 20 were removed. Finally, trimmed and filtered reads shorter than 60 bp were also removed. The filtered reads were mapped to the *A. nigrocauda* genome (data unpublished) using HISAT v2.0.0 (Kim et al., 2015) with default parameters. The program htseq-count within HTSeq v0.12.4 (Anders et al., 2015) was used to obtain the count as BAM files mapping to each gene.

Differentially expressed genes (DEGs) were identified using a negative binomial distribution model by analyzing the read coverage data included in DESeq2 in the R software (Love et al., 2014). Only genes with twofold expression change and *p*_adj_ < 0.05 were treated as authentic DEGs.

### 2.4 GO and KEGG Enrichment Analyses

The enrichment analyses were performed using GeneSCF v1.1-p2 (Subhash and Kanduri, 2016). For gene ontology (GO) and Kyoto Encyclopedia of Genes and Genomes (KEGG) enrichment analyses, “−org = zfin” and “−org = dre” were used as parameters. The GO and KEGG databases were updated in July 2020 (http://www.genome.jp/kegg/).

### 2.5 Gene Co-Expression Network Construction With WGCNA

To find the relationship between the body weight and identified DEGs, the WGCNA was conducted using the WGCNA package v1.70-3 following a previously published procedure (Langfelder and Horvath, 2008). For this analysis, we only used the top 50% of genes with large variance to check the presence of outliers. The expression correlation coefficients of these genes were used as a suitable soft threshold using a scale-free topology model. Next, all gene models with similar expression patterns were identified using the gene cluster dendrogram with dynamic tree cut algorithm (minModuleSize = 30 and mergeCutHeight = 0.25) (Wang et al., 2018a). Finally, we used all genes within each module to assess their correlation with body weight. Modules with a high correlation value and *p* < 0.05 were considered as body weight-related modules.

## 3 Results

### 3.1 RNA-Seq of Liver and Brain Tissues From Female and Male *A. nigrocauda*

Liver and brain tissues were isolated from six female and six male fish aged 18 months and reared in the same pond. The difference in the body weight between males and females was found to be significant using the *t*-test (*p* = 3.618e-06; Supplementary Table S1), proving that *A. nigrocauda* exhibited SSD. We extracted the mRNA from tissues and used it to construct a library, which was subsequently sequenced on an Illumina NovaSeq platform. The liver and brain tissue samples had 26.47 and 25.03 million read pairs on average, respectively (Table 1). The percentage of clean reads in raw reads ranged from 93.49 to 95.08 in the liver tissue and from 93.29 to 95.18 in the brain tissue (Table 1). After mapping filtered reads to the *A. nigrocauda* genome, the average overall alignment rate was 88.98 and 84.56 for liver and brain, respectively. Among the mapped reads, 72.12 and 76.78% reads on average were uniquely mapped to the liver and brain, respectively (Table 1).

### 3.2 Gene Expression Analysis for Liver and Brain Tissues

Based on the reference annotation, we found that 19,080 genes were expressed in at least six samples in the liver. Among these, 865 genes exhibited extremely high expression with a Fragments per Kilobase of transcript per Million (FPKM) greater than 100 in the six samples. The KEGG enrichment analysis showed that these genes were enriched [false discovery rate (FDR) < 0.05] in 42 pathways, including oxidative phosphorylation, PPAR signaling pathway, fatty acid degradation, and other pathways involved primarily in liver metabolism (Supplementary Table S2). Similarly, 26,672 genes were expressed in at least six brain samples, of which 1,507 genes were highly expressed and enriched in 23 KEGG pathways, such as ribosome, spliceosome, and proteasome (Supplementary Table S3).

**FIGURE 1:**
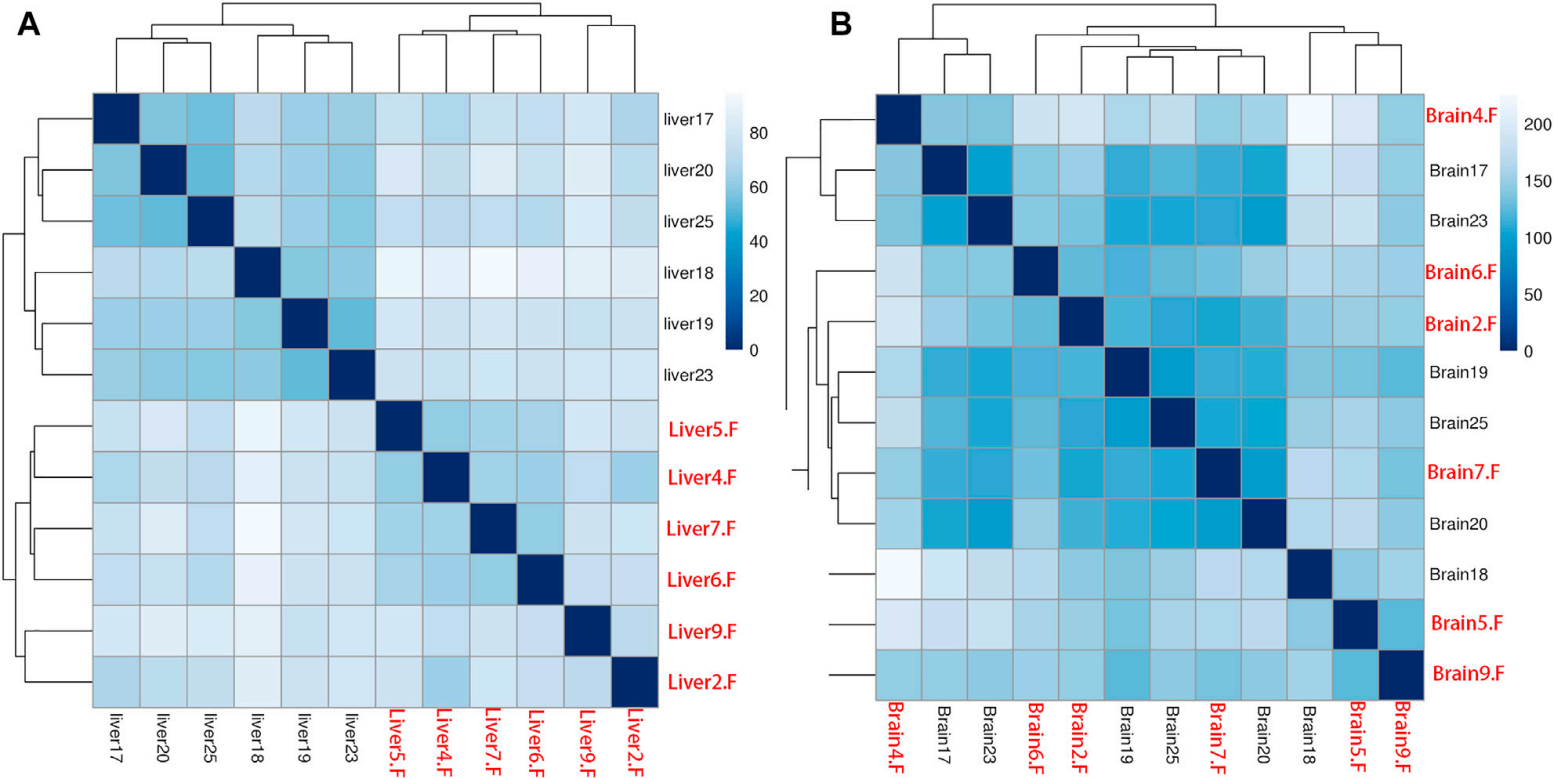
Person’s correlation for all samples in liver **(A)** and brain **(B)** tissues based on gene expression. All female samples are colored with red and all male samples are colored with black.

After quantification of all genes in the two tissues, we performed sample clustering to assess the quality of our RNA-seq data. No anomalies were observed in the Euclidean distance matrix in the liver tissue when compared with samples’ body weight (Figure 1A). However, we found samples from brain tissue were mixed and did not group by their body weight (Figure 1B).

### 3.3 Differential Gene Expression Analysis in the Liver and Brain

**FIGURE 2:**
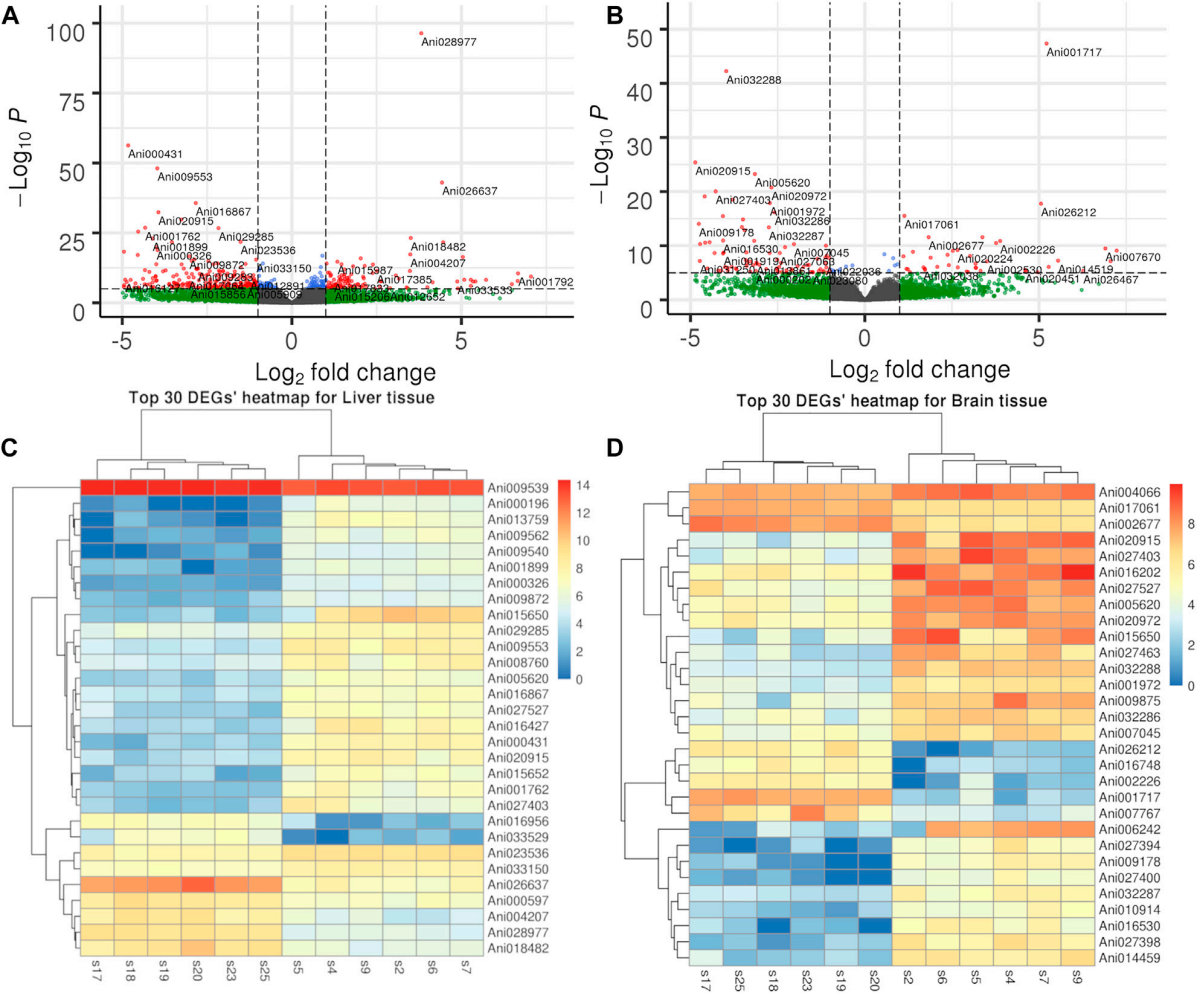
Volcano plot and heatmap of DEGs in liver and brain tissues. **(A)**. Volcano plot of DEGs in liver tissue. **(B)**. Volcano plot of DEGs in brain tissue. **(C)**. Hierarchical clustering of top 30 DEG in liver tissue. **(D)**. Hierarchical clustering of top 30 DEG in brain tissue.

**FIGURE 3:**
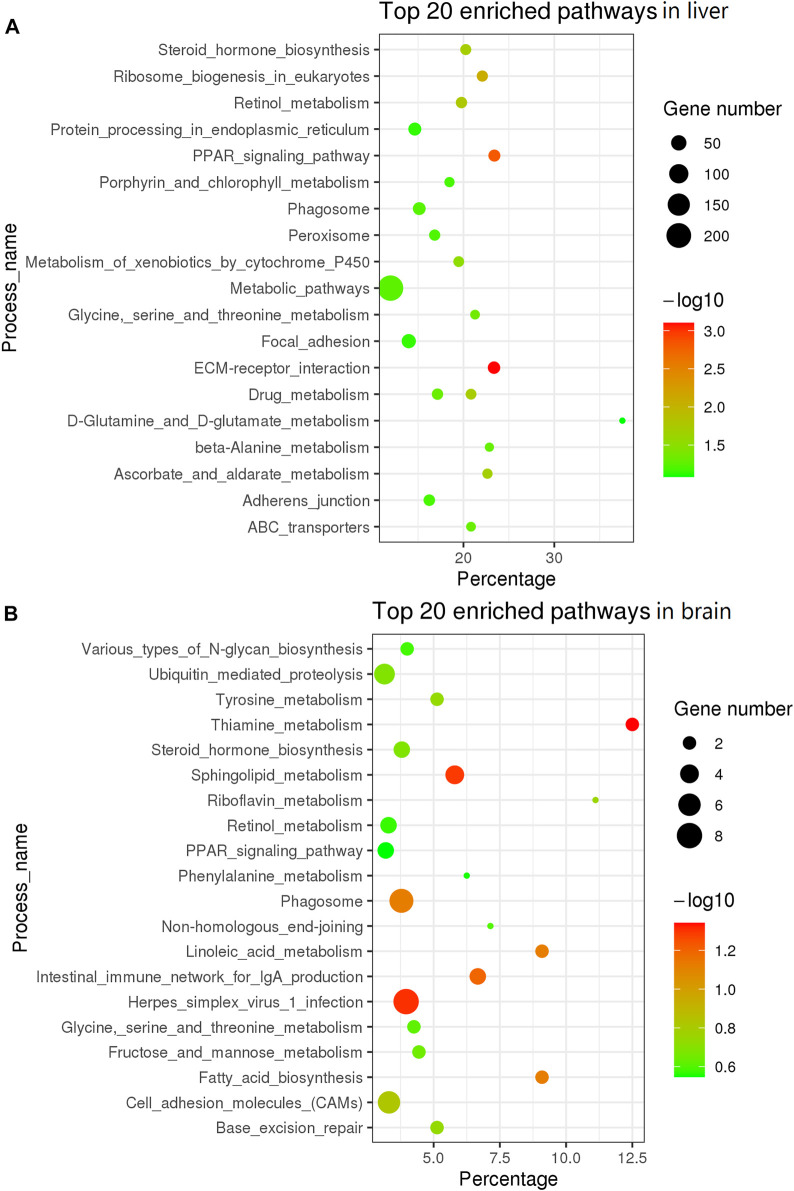
KEGG enrichment analysis of top 20 pathway in liver and brain tissue. **(A)** KEGG in top 20 pathways in liver. **(B)** KEGG in top 20 pathways in brain tissue.

To further understand the difference in SD between the liver and brain tissues of the two sexes, a comparative transcriptome analysis was conducted. A total of 2,395 DEGs were detected in the liver tissue, of which 1,401 genes were upregulated and 994 genes were downregulated in the female liver when compared with the male liver (Figure 2A; Supplementary Table S4). Moreover, the top 30 DEGs in the liver tissue were hierarchically clustered to obtain a comprehensive view of all DEGs between the two sexes (Figure 2C). In these DEGs, *mhc1uka* has been reported to show a much higher expression in testes than in ovary in zebrafish at 21dpf (Yan et al., 2019). And psmb4’s overexpression enhanced the cell growth and viability of breast cancer and ovarian cancer cells (Liu et al., 2016; Wang et al., 2018b). Col6a1, which belongs to Collagen type VI (COL6) family, served an important role in regulating cell proliferation, apoptosis and invasion (Chen et al., 2013). We next used the geneSCF tool to perform the GO enrichment analysis and found that the GO terms related to cholesterol homeostasis were over-represented: cholesterol homeostasis (GO:GO:0042632), triglyceride homeostasis (GO:0070328), and triglyceride metabolism (GO:0006641). In addition, the KEGG enrichment analysis was performed and the top 20 pathways are presented in Figure 3A. Among these, the PPAR signaling pathway and ECM-receptor interaction were on the top of the list.

In the brain tissue, we found only 457 DEGs, of which 293 genes were upregulated and 164 genes were downregulated in the female tissue when compared to the male tissue (Figure 2B; Supplementary Table S5). The top 30 DEGs in the brain tissue were hierarchically clustered and show the same pattern as that in liver tissue (Figure 2D). The KEGG enrichment analysis of these DEGs revealed that they were largely enriched in steroid hormone biosynthesis, PPAR signaling pathway, and cell adhesion molecules (CAMs) (Figure 3B).

We also found 152 DEGs presented in both tissues, among which there are many growth-related genes showed much higher expression level in female samples, such as *atriaid* (positive regulation of osteoblast proliferation), *cetp* (triglyceride transport), and *pm20d1.1* (amide biosynthetic process). Besides, we also found genes involved in energy supply and oxygen circulation were on the top of enriched DEGs, as a key metabolic organelle, mitochondria is the core for energy supply in cells and therefore is key to cell growth (Ryu et al., 2020). Apart from the evenly distributed protein coding genes, these DEGs showed a totally different pattern that some chromosomes have more DEGs than others. We found the density of DEGs on LG1 and LG20 were much higher than the rest (Table 2). Though DEGs on LG20 were not located in sex-specific region of *A. nigrocauda,* they were only several megabases away from that region (Sun et al., 2021). For DEGs on LG1, they were not randomly distributed, either. Previous study didn’t find any sex-specific region on LG1, but the DEGs were mostly located in a 5 Mb regions.

**TABLE 2:** The uneven distribution of DEGs on each chromosome in brain and liver tissues.

	Length	DEG in brain	DEG in liver	protein-coding num	gene density per Mb
Superscaffold1	66382954	58	214	2347	35.35546189
Superscaffold2	62144506	31	152	2162	34.78988151
Superscaffold3	56867972	22	112	1504	26.44722411
Superscaffold4	57515027	14	96	1679	29.19237089
Superscaffold5	41970900	8	57	1174	27.97176139
Superscaffold6	43753008	17	98	1439	32.8891673
Superscaffold7	51368025	32	155	1713	33.34759318
Superscaffold8	48371291	25	116	1550	32.04380053
Superscaffold9	43188871	23	115	1331	30.81812442
Superscaffold10	40104110	14	96	1360	33.91173623
Superscaffold11	44045269	12	81	1433	32.53470878
Superscaffold12	42678128	29	118	1333	31.23379732
Superscaffold13	44203473	11	76	1415	32.0110594
Superscaffold14	40391094	10	98	1224	30.30371002
Superscaffold15	32058659	19	74	1087	33.90659603
Superscaffold16	41350092	9	68	1265	30.59243496
Superscaffold17	36596543	10	76	1274	34.81203129
Superscaffold18	36063290	10	87	1155	32.02702804
Superscaffold19	40936703	14	76	1252	30.58380153
Superscaffold20	36861994	31	107	1220	33.09641904
Superscaffold21	41197416	24	102	1417	34.39536111
Superscaffold22	31363827	12	62	1021	32.55342532
Superscaffold23	37215343	12	76	1121	30.1219849
Superscaffold24	32612968	9	74	975	29.89608306

### 3.4 Construction of Gene Co-expressed Network Using WGCNA

**FIGURE 4:**
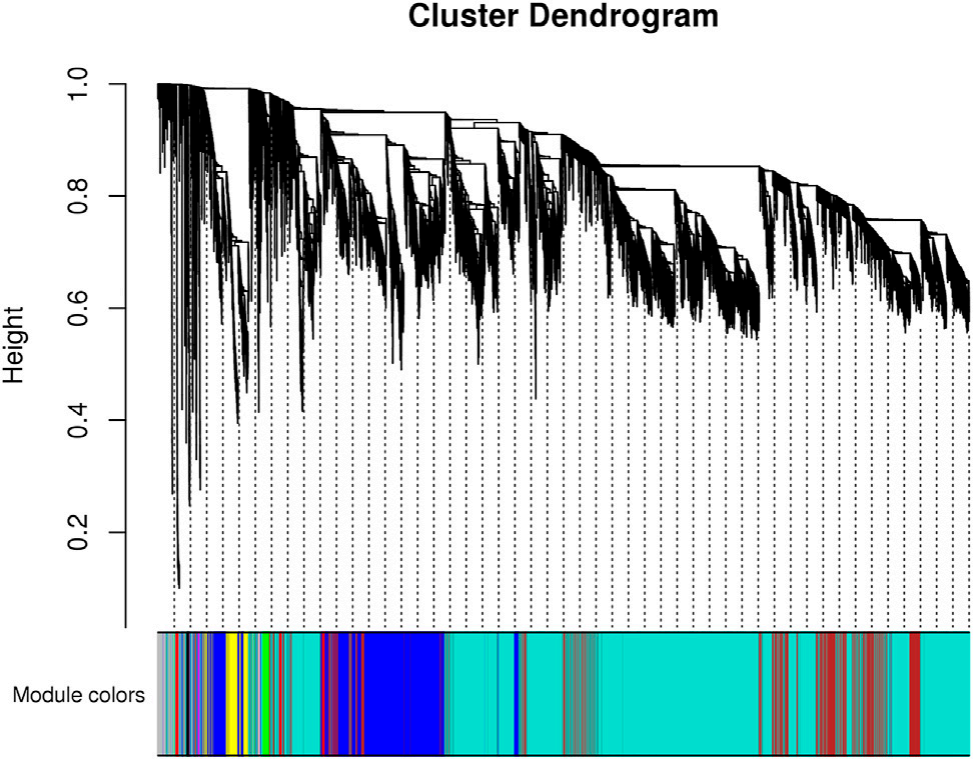
The gene cluster dendrogram constructed by all genes’ correlation coefficients. The vertical distance of the line shows the distance between different genes.

**FIGURE 5:**
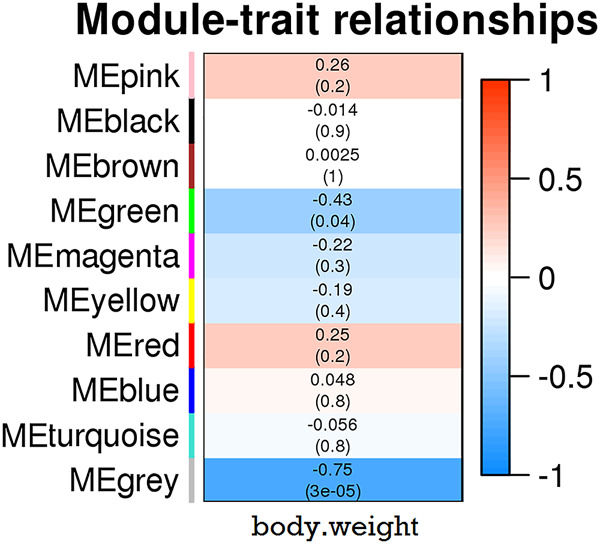
The relationship between 10 modules and growth traits in samples. The eigengene in each module was calculated and shown in each row. The color bar indicates correlation value from low (blue) to high (red).

To identify the genetic modules correlating with body weight in the transcriptome data, we used WGCNA to construct a co-expressed gene network using genes whose variances were among the top 50% of all annotated genes. Next, only 16,803 genes were used for subsequent analysis. Based on the power estimation of these genes, a gene cluster dendrogram was constructed with a power value of 16 (Figure 4). Afterward, 10 modules were identified with gene numbers ranging from 31 to 9,935 (Supplementary Table S6). To identify body weight-correlated modules, module–phenotype correlation was conducted, which showed that only the green module exhibited a significant correlation (*p* = 0.04) (Figure 5).

For the KEGG pathway enrichment analysis, 168 genes within green module were significantly enriched in protein processing in the endoplasmic reticulum (dre04141, FDR = 1.74E-12), ribosome biogenesis in eukaryotes (dre03008, FDR = 6.95E-07), and protein export (dre03060, FDR = 8.75E-4). In the GO biological process, these genes were enriched in protein transport (GO:0015031), protein glycosylation (GO:0006486), and other protein metabolic processes (FDR < 0.05).

## 4 Discussion

The phenomenon of SSD is reported in several fish species and forms the genetic foundation for monosex breeding in aquaculture (Mei and Gui, 2015). The SSD in fish is primarily caused by differences in gene expression, which recently have expanded from the involvement of sex-chromosome to autosomes (Parsch and Ellegren, 2013). However, the genetic mechanism is still unclear in several fish species. We performed a comparative transcriptome analysis in both sexes of *A. nigrocauda* using liver and brain tissues. According to a previous report, biological replicate for each group is a prerequisite for conducting comparative RNA-seq analysis. In addition, the use of a higher number of replicates results in the identification of more authentic DEGs (McIntyre et al., 2011; Schurch et al., 2016). We found that the majority of transcriptome analyses regarding SSD were based on only two or three replicates in each group (Wang et al., 2018a; Ryu et al., 2020), leading to the loss of numerous authentic DEGs and several false-positive genes (Fang and Cui, 2011). To overcome these disadvantages, we included six samples in each group (Figure 1), which we believed to be the best arrangement in an SSD-related study to date. In addition, sample clustering revealed a distinct expression pattern in different sexes (Figure 1). Furthermore, the high quality of raw sequencing reads and mapping ratio proved the high quality of RNA-seq data (Table 1).

We selected sexually matured *A. nigrocauda* to study the gene expression patterns in liver and brain tissues. The majority of the previous studies on SSD in other fishes used only one tissue (Huang et al., 2018; Ryu et al., 2020); only a few have studied the expression patterns in different tissues simultaneously (Wang et al., 2018a), providing a definite genetic pattern regulating the body size. The gene expression is tissue-specific, with different tissues showing distinct expression patterns in a single study. Therefore, multi-tissue transcriptome analyses are attracting attention because of their high resolution in deciphering biological or genetic regulation (Fu et al., 2019).

The hypothalamic–pituitary–adrenal (HPA) axis is critical for fish growth and sex-related actions (Toews et al., 2021). The liver greatly affects ontogenesis by integrating diverse endocrine signals that regulate body growth and sexual maturity in fish. In addition, hepatocytes respond to alterations in the metabolism and physiological state. Several hepatic genes regulating these responses are hormone-dependent, which in turn are controlled by factors such as GH and IGF1. Furthermore, other glucocorticoid hormones are known to regulate hepatic gluconeogenesis and glucose availability from the brain and other tissues (Loria et al., 2009). We demonstrated that several DEGs in the liver, such as *ghrb*, *igfbp1b*, *igfbp6b*, and *igf2bp2b*, belonged to the GH/IGF pathway and exhibited a sex-specific expression pattern*.* This pattern was the same as that observed in *Cynoglossus semilaevis*, which also exhibits female-biased SSD (Wang et al., 2018a). In addition to the GH/IGF pathway, its downstream regulated Jak2/Stat5b pathway significantly affects the body weight of fish, too (Rotwein, 2012). For example, Stat5b regulates the male-biased genes in the liver, whereas Stat5a primarily regulates the female-biased gene in the liver (Lamba et al., 2014; Huang et al., 2018). In this study, although *stat5b* did not show a significant expression pattern in the liver and brain tissues (*p* = 0.79 and *p* = 0.99, respectively), *stat5a* was significantly expressed in the liver (FDR = 0.0279). In addition, both Stat5b and Stat5a regulate SD by controlling the activity of hepatic cytochromes P450 (CYPs) (Lamba et al., 2016). CYPs have been reported to exhibit a sexual dimorphism expression pattern (Xiong et al., 2015). We found cyp2ad3, cyp2p7, cyp2k6, cyp1c1, cyp2k16, and cyp2x7 displayed a substantially higher expression in the female liver than in the male liver. However, the expression of cyp11c1, cyp27b1, cyp3a65, and cyp2k21 weas higher in the male liver than in the female liver.

Numerous neuroendocrine factors that control the reproductive axes are secreted in the brain tissue. We found only 457 DEGs between female and male brains compared to a considerably higher number in the liver. However, this finding was similar to that obtained in *C. semilaevis* brain and could be attributed to the fact that the crucial stage for feminization or masculinization of the fish brain might begin during the early stage of its development (Wang et al., 2018a). When fishes reach sexual maturity, the brain’s differentiation in both sexes is completed due to exposure to different sex steroid hormones. Previous studies have reported *Pomc* as a candidate gene for SSD in tilapia (Wan et al., 2021). Its high expression in the female tilapia brain has been associated with the slow growth of the fish (Wan et al., 2021). We found that the expression of *Pomc* waes considerably higher in the male brain than in the female brain, a pattern same as that observed in tilapia.

WGCNA analysis has been used in many transcriptome studies to found genes or pathways that correlated with specific phenotype (Tao et al., 2018; Huang et al., 2020). In this study, we used WGCNA to find modules associated with sexually size dimorphism and only one module was significantly associated with it. Genes within the module has been reported to contributed to the energy metabolism and protein synthesis (Supplementary Table S6). This pattern is the same as SSD in olive flounder (Ryu et al., 2020) as mitochondria is the key organelle for cell energy supply and protein is the key substance for cell growth.

## 5 Conclusion

In summary, we used RNA-seq to study the distinct expression pattern of genes in the liver and brain of female-biased *A. nigrocauda.* The number of DEGs was considerably higher in the liver tissue than in the brain tissue, which could be attributed to the following reasons. First, we used six samples in each group of each tissue; thus, a large number of samples provided a statistically concrete ground for identifying authentic DEGs. Second, the ongoing protein and lipid metabolism in the liver contribute to different growth rates between the sexes at sexual maturity versus the completely differentiated brain exposed to sex steroids. Altogether, our results revealed a transcriptome pattern of liver and brain tissues in fish with SSD. In addition, the WGCNA of two tissues allowed us to study the regulatory network involved in fish growth.

## Data Availability

The datasets generated for this study can be found in the NCBI https://www.ncbi.nlm.nih.gov/search/all/?term=PRJNA762193.
